# Ferroelectric Interfaces for Dendrite Prevention in Zinc‐Ion Batteries

**DOI:** 10.1002/smll.202403555

**Published:** 2024-09-15

**Authors:** Xueqing Hu, Bastola Narayan, Nibagani Naresh, Iman Pinnock, Yijia Zhu, Xiaopeng Liu, Tianlei Wang, Bing Li, Ivan P. Parkin, Buddha Deka Boruah

**Affiliations:** ^1^ Institute for Materials Discovery (IMD) University College London (UCL) London WC1E 7JE UK; ^2^ Department of Mechanical Engineering University of Bath Bath BA2 7AY UK; ^3^ Department of Chemistry University College London (UCL) London WC1H 0AJ UK

**Keywords:** dendrite suppression, ferroelectric, ohmic contact, zn anodes, zn‐ion batteries

## Abstract

Aqueous rechargeable zinc‐ion batteries (ZIBs) are increasingly recognized as promising energy storage systems for mini‐grid and mini‐off‐grid applications due to their advantageous characteristics such as high safety, affordability, and considerable theoretical capacity. However, the long‐term cycling performance of ZIBs is hampered by challenges including the uncontrolled dendrite formation, the passivation, and the occurrence of the hydrogen evolution reaction (HER) on the Zn anode. In this study, enhancing ZIB performance by implementing oxide material coatings on Zn metal, serving as a physical barrier at the electrode‐electrolyte interfaces to mitigate dendrite growth and suppress the HER is concentrated. Specifically, the mechanisms through which the n‐type semiconductor TiO_2_ coated Zn anode establishes ohmic contact with Zn, and the high‐dielectric BaTiO_3_ (BTO) coated Zn anode fosters Maxwell‐Wagner polarization with ferroelectric properties, significantly inhibiting dendrite growth and side reactions, thereby resulting in a highly stable Zn anode for efficient aqueous ZIBs is explored. This advanced BTO/Zn electrode demonstrates an extended lifespan of over 700 h compared to bare Zn and TiO_2_/Zn anodes. Additionally, full‐cell aqueous ZIBs incorporating BTO/Zn//VO_2_ (B) batteries exhibit superior rate capabilities, high capacity, and sustained cycle life.

## Graphical abstract

The development of artificial solid‐electrolyte interphase on zinc anodes offers a promising solution to mitigate dendrite formation and inhibit hydrogen evolution reactions in high‐performance aqueous zinc‐ion batteries. By exploring the application of barium titanate coatings on zinc anodes, the high‐dielectric barium titanate layer creates both an ohmic contact interface and establishes electric dipoles at the interface. These electric dipoles can be oriented using an external electric field, and during charge–discharge cycles, the synergistic effects of the ohmic contact and aligned electric dipoles efficiently guide the movement of zinc ions.

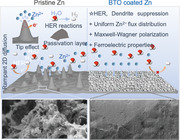

## 1 Introduction

Given the considerable challenges posed by environmental issues and energy crises resulting from overreliance on fossil fuels, the global energy landscape is currently undergoing substantial transformations. Central to the advancement of future energy systems is energy storage technology, which holds the potential to significantly impact upcoming energy infrastructure and security. Within the energy storage system, the rechargeable battery plays a crucial role in the continuum of energy storage technology. Among rechargeable batteries, lithium‐ion batteries have emerged as the market leader and have found widespread use in modern technology, from handheld electronics to EVs. Their popularity is attributed to their lightweight nature, high energy density, and long‐term stability.^[^ ^1^ ^]^ However, concerns about the availability of materials, cost competitiveness, and safety indicate that lithium‐ion batteries may not be the optimal choice for environmentally sustainable and cost‐effective alternatives, particularly in the context of mini‐grid and mini off‐grid energy storage solutions. As a result, the rapidly advancing field of new energy storage systems has led to an increased demand for innovative batteries that encompass characteristics such as energy density, safety, cost‐effectiveness, and eco‐friendliness. Here, aqueous ZIBs have recently become an important part of electrochemical energy storage.^[^ ^2^ ^]^ High‐capacity oxide‐based materials (V‐based and Mn‐based) are considered potential cathodes with metallic Zn anodes, creating a promising electrode configuration for ZIBs.^[^ ^3^ ^]^ The properties of metallic Zn anodes, including high theoretical capacity (≈820 mAh g^−1^or ≈5855 mAh cm^−3^), low redox potential (−0.76 V vs standard hydrogen electrode), and suitability for deployment in aqueous electrolytes enable a two‐electron transfer process during redox reactions, contributing to the attainment of high‐energy density in ZIBs.^[^ ^4^ ^]^

While Zn metal stands out as an ideal choice for the anode in ZIBs, it faces a significant challenge in the form of dendrite growth when exposed to aqueous electrolytes.^[^ ^5^ ^]^ This challenge gives rise to unfavorable side reactions, including the formation of Zn dendrites, the HER, passivation, and corrosion, ultimately leading to a decrease in capacity over extended cycling periods.^[^ ^5^, ^6^ ^]^ To address these issues, various strategies have been explored. These include the application of artificial surface coatings on Zn anodes, the manipulation of the electrolyte composition, and the development of advanced separator designs.^[^ ^6^ ^]^ Among these approaches, the application of artificial coatings directly onto the Zn surface emerges as an easy‐to‐process, practical, and direct solution that can be implemented on an industrial scale.^[^ ^7^ ^]^

These coatings create a barrier between the electrolyte and the Zn electrode, effectively reducing the chance of direct water‐Zn contact. This minimizes the occurrence of the HER and enhances the kinetics of Zn^2+^ plating and stripping, while also ensuring a uniform electric field distribution. Various oxide materials, such as TiO_2_,^[^ ^8^ ^]^ ZnO,^[^ ^9^ ^]^ CeO_2_,^[^ ^10^ ^]^ BaTiOBTO),^[^ ^11^ ^]^ Nb_2_O_5_,^[^ ^12^ ^]^ ZrO_2_,^[^ ^13^ ^]^ and others, have been utilized as artificial layers on Zn anodes due to their chemical and thermal stability. Materials such as ZnO, TiO_2_, and Nb_2_O_5_ have demonstrated the ability to form ohmic contacts with Zn, facilitating efficient electron transfer between the Zn and oxide layers.^[^ ^9^, ^12^ ^]^ This results in a uniform electric field distribution, promoting consistent Zn^2^⁺ plating and stripping. Furthermore, the polar surfaces of these oxides assist in the desolvation of solvated Zn^2^⁺ ions, which helps reduce HER and enhances the stability of the Zn anode.^[^ ^9^ ^]^ High‐dielectric materials like BTO exhibit both Maxwell‐Wagner and ferroelectric effects, characterized by a high dielectric constant in metal‐oxide/perovskite structures.^[^ ^14^ ^]^ These properties have been shown to effectively suppress non‐uniform Zn dendrite formation. The Maxwell‐Wagner polarization, commonly observed in high‐dielectric coatings, generates a uniform electric field flux at the Zn anode surface and aids in creating a balanced ion distribution. Additionally, BTO materials display space charge polarization during Zn^2^⁺ plating when an electric field is applied. However, a deeper understanding is needed to determine whether n‐type materials that form ohmic contacts with Zn or high‐dielectric materials that promote the Maxwell‐Wagner effect with ferroelectric properties are more effective at suppressing Zn dendrite growth and improving cycling stability in ZIBs, as sequential studies on this comparison remain scarce.

In this research, we examined the effects of applying TiO_2_ and BTO coatings on Zn anodes to suppress dendrite formation in ZIBs. The primary goal was to assess how these coatings, which either form Ohmic contacts with Zn or utilize high‐dielectric constant materials that promote the Maxwell‐Wagner effect with ferroelectric properties, influence dendrite growth. Interestingly, our in‐depth analysis revealed that, even with identical coating thicknesses and particle sizes, Zn anodes coated with BTO exhibited a significantly greater ability to suppress Zn dendrites compared to those coated with TiO_2_ and uncoated Zn anodes. As expected, when subjected to full cell of BTO‐coated Zn anodes tests against VO_2_ (B) cathodes, the cells exhibited better charge storage performance, including enhanced cycling stability and rate capability, compared to Zn//VO_2_ (B) cells.

## 2 Result and Discussion

**Figure 1:**
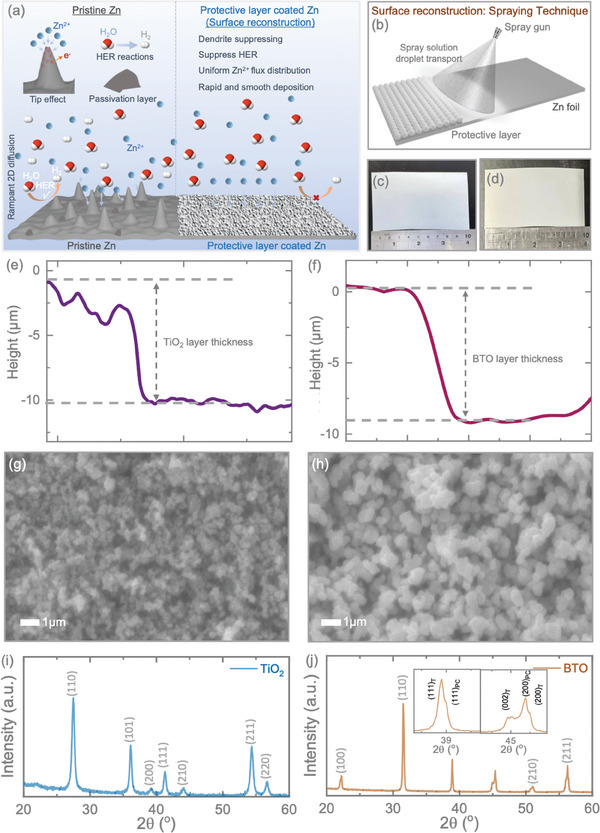
a) Schematic illustrating dendrite growth, passivation, and HER on pristine Zn, while the application of a protective layer suppresses these processes, providing long‐term cycling stability when used as anode materials in ZIBs. b) Schematic depicting the spray processing technique for surface reconstruction, followed by the spraying of respective materials onto Zn anodes. Digital images showing the spray‐coated c) TiO_2_ and d) BTO onto Zn metal foils. e, f) Depth profile of coated TiO_2_ and BTO onto Zn. g, h) SEM image of used TiO_2_ and BTO nanoparticles. XRD patterns of i) TiO_2_ and j) BTO nanoparticles.

The schematic illustration of the Zn dendrite growth mechanism, passivation, HER reaction, and protective high‐dielectric layer coating on the Zn anode is displayed in **Figure** **1**. The utilization of pristine Zn as an anode in aqueous electrolytes for ZIBs is impeded by dendrite growth, resulting from the uneven deposition of Zn^2+^ due to the tip effect (Figure 1a), passivation layers, and the introduction of the HER, which diminishes the cycling stability of ZIBs. However, the surface reconstruction of Zn anodes through the application of an electrochemically stable coating mitigates these challenges and thereby enhances the long‐term cycling stability of Zn anodes. Herein, we have investigated by constructing an Ohmic layer interface on the Zn surface via deposition of protective layer TiO_2_ on the Zn anode or the role of high dielectric BTO on Zn surface via creating space charge distribution between the electrode and electrolyte interface. Surface reconstruction represents a straightforward approach to suppressing dendrite growth on Zn anodes and is commercially viable. The use of spray processing techniques (Figure 1b) proves to be a robust and rapid method for coating materials onto Zn anodes to serve as protective layers, offering flexibility in controlling the thickness of the coating materials. Recognizing the advantages of spray processing techniques, we employed this approach to coat TiO_2_ and BTO nanoparticles uniformly onto Zn foil to serve as anodes. Figure 1c–d depict digital images of the samples, demonstrating the uniform application of TiO_2_ and BTO onto Zn foil over a large area (10 × 10 cm^2^). The coating thickness of TiO_2_ and BTO was optimized by controlling the spraying time, with optimum performance observed at a thickness of ≈10 µm, as confirmed by the height profiles in Figure 1e,f. Figure 1g,h present scanning electron microscope (SEM) images of the utilized TiO_2_ (size = 50–100 nm) and BTO (size = 50–100 nm) nanoparticles. The EDS mappings of the samples are included in the Supporting Information (Figure S1, Supporting Information). The X‐ray diffraction pattern (XRD) of the TiO_2_ and BTO nanoparticles validates their crystallinity. The XRD pattern of TiO_2_ (Figure 1i) confirms rutile TiO_2_, with diffraction peaks at 27.33°, 36.09°, 41.23°, 44.14°, 54.36°, 56.46°, and 56.46°, corresponding to the (110), (101), (111), (210), (211), and (220) crystal planes, respectively, in accordance with the standard card (JCPDS No. 21–1276). Whereas XRD pattern of the BTO nanoparticles shows a pure perovskite phase without any secondary phases (Figure 1j). The strong Bragg's reflections were observed ≈2θ of 22.2°, 31.5°, 38.9°, 45.4°, 51°, and 56.3° corresponds to the (100), (110), (111), (200), (210), and (211) pseudo‐cubic miller indices of BTO (JCPDS number #892 475).^[^ ^15^ ^]^ The inset shows the magnified (111) and (200) reflections. A clear splitting of (200) suggests the presence of a ferroelectric tetragonal structure with (P4mm) crystal symmetry. A strong singlet‐like peak can be seen in between the 200 tetragonal splitting corresponds to the ferroelectric pseudocubic phase.

**Figure 2:**
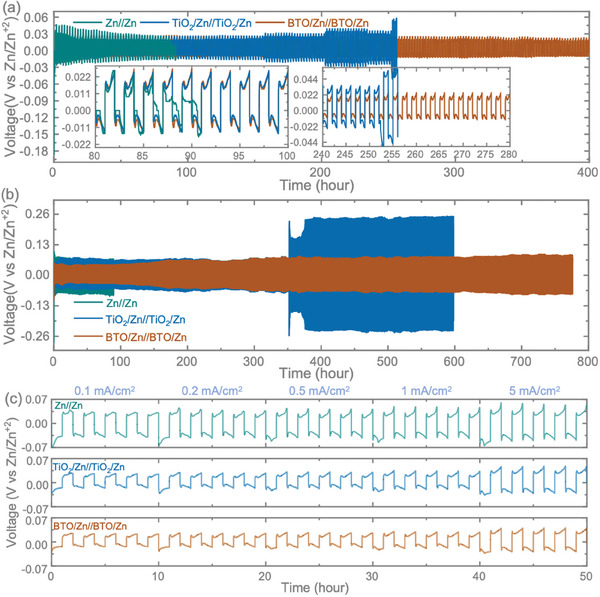
Voltage profiles obtained from a symmetrical cell using Zn, TiO_2_/Zn, and BTO/Zn anodes were investigated at areal currents of a) 1 mA cm^−2^ (at 1 mAh cm^−2^) and b) 5 mA cm^−2^ (at 1 mAh cm^−2^). c) Assessment of the rate performances of the symmetrical cells under various areal currents, including 0.1 mA cm^−2^ (at 0.1 mAh cm^−2^), 0.2 mA cm^−2^ (at 0.2 mAh cm^−2^), 0.5 mA cm^−2^ (at 0.5 mAh cm^−2^), 1 mA cm^−2^ (at 1 mAh cm^−2^), and 5 mA cm^−2^ (at 5 mAh cm^−2^).

To evaluate the cyclic stability of the Zn^2+^ stripping/plating process in electrodes, including pristine Zn, TiO_2_‐coated Zn (TiO_2_/Zn), and BTO‐coated Zn (BTO/Zn) anodes, symmetric cells were assembled using these electrodes, denoted as Zn//Zn, TiO_2_/Zn//TiO_2_/Zn, and BTO/Zn//BTO/Zn, respectively. To understand the Zn^2+^ plating/stripping behavior on pristine Zn and coated Zn, cycling performances were measured under different areal currents, and voltage profiles were recorded. In **Figure** **2**a, voltage profiles of symmetric cells at a constant areal current of 1 at 1 mAh cm^−2^ are depicted. The bare Zn//Zn cell exhibits an average voltage hysteresis of ≈72 mV. In contrast, TiO_2_/Zn//TiO_2_/Zn and BTO/Zn//BTO/Zn demonstrate lower hysteresis values, ≈47 mV each, indicating an improvement compared to their pristine counterparts. At a higher areal current of 5 mA cm^−2^ at 5 mAh cm^−2^, BTO/Zn//BTO/Zn exhibits a relatively lower and stable voltage profile for long‐term cycling, with an average voltage hysteresis of 91 mV. This is in contrast to Zn//Zn and TiO_2_/Zn//TiO_2_/Zn cells, which display hysteresis values of 187 and 92 mV, respectively (Figure 2b). The reduced voltage hysteresis suggests improved Zn^2+^ transfer kinetics, leading to a more stable Zn^2+^ plating/stripping process and consequently extending the cycling life.^[^ ^16^, ^17^ ^]^ To delve deeper into these characteristics, SEM images of cycled electrodes (at 1 mA cm^−2^) were captured to understand the surface morphologies during Zn^2+^ ion stripping/plating. As depicted in Figure S2 (Supporting Information), SEM images taken after the experiment display a rough surface with pronounced dendrites on the untreated Zn electrode, contrasting with the coated electrodes. However, obtaining clear morphological images proves challenging, particularly with symmetric cells in the coin cell configuration, as the conventional glass fiber separator adheres to the anode surface when cycling and is difficult to remove. To ensure fair morphological analysis, we devised a specialized cell design for direct dendrite monitoring in the absence of a separator, capturing surface morphology while Zn^2+^ ions plate onto the anodes (see further). Moreover, voltage profile tests at different areal currents ranging from 0.1 to 5 mA cm^−2^, as depicted in Figure 2c, further confirm lower voltage hysteresis profiles in BTO/Zn//BTO/Zn than TiO_2_/Zn//TiO_2_/Zn and Zn//Zn, where higher values are observed in Zn//Zn at each areal current compared to that of coated electrodes.

**Figure 3:**
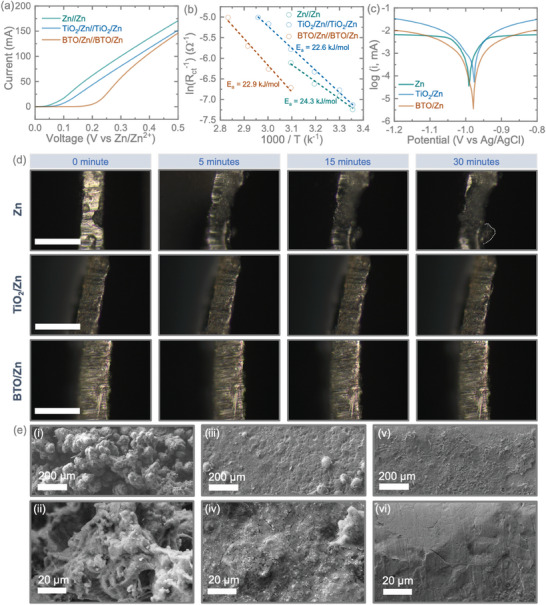
a) Linear sweep voltammetry (LSV) results of symmetrical cells tested at 0.5 mV s^−1^. b) Arrhenius plots and a comparison of activation energies for Zn, TiO_2_/Zn, and BTO/Zn anodes. e) Tafel plots of anodes tested in a three‐electrode system at 0.5 mV s^−1^ using AgCl and platinum as reference and counter electrodes. d) In situ optical images of the anodes, specifically Zn, TiO_2_/Zn, and BTO/Zn, were captured at various time intervals ranging from 0 to 30 min during the Zn deposition process. These assessments were conducted using symmetric cell configurations at a current density of 10 mA cm^−2^. The scale of the images is set at 500 µm. e) SEM images of the anodes of i, ii) pristine Zn, iii, iv) TiO_2_/Zn and v, vi) BTO/Zn at low and high magnifications after 30 min Zn^2+^ plattings in d).

**Figure** **3**a depicts the linear sweep voltammetry (LSV) of symmetric cells. Interestingly, it is observed that the pristine Zn exhibited a relatively larger current compared to TiO_2_/Zn and BTO/Zn anodes under the same voltage. Consequently, coated samples exhibit higher Oxygen Reduction Reaction (ORR) activity under the same voltage, providing a lower current than that of the pristine sample. Additionally, we conducted Electrochemical Impedance Spectroscopy (EIS) on symmetric cells at different temperatures to evaluate the activation energies of the anodes. As depicted in the Arrhenius plots in Figure 3b, ln(R_ct_^−1^) is linear as a function of 1000/T. The absolute value of the slope of BTO/Zn and TiO_2_/Zn is smaller than that of Zn. The calculated activation energies are 22.6 and 22.9 kJ mol^−1^ for BTO/Zn and TiO_2_/Zn, respectively, which are smaller than the value for Zn (24.3 kJ mol^−1^). Coating with BTO and TiO_2_ improves interfacial charge transfer kinetics, with BTO/Zn providing a better value. Furthermore, we assessed the corrosion resistance of the samples using a Tafel curve (Figure 3c). Lower exchange current densities result in greater corrosion resistance. The BTO/Zn and TiO_2_/Zn anodes showed the lowest exchange current densities of 1.7 and 1.2 mA cm^−2^ compared to bare Zn (3.5 mA cm^−2^), indicating the lowest corrosion activity of BTO/Zn and TiO_2_/Zn.^[^ ^18^ ^]^ Subsequent to these experiments, we conducted *in situ* monitoring of dendrite growth on pristine Zn anodes as well as on anodes coated with TiO_2_ and BTO. For these assessments, we utilized a specially designed symmetric cell for real‐time monitoring of dendrite growth, as opposed to using a coin cell (refer to Figure S3, Supporting Information). Zn^2+^ plating was carried out by applying a specific current of 10 mA cm^−2^ to the anodes, and optical microscopic images were captured from the side of the electrode at various plating time intervals (0, 15, and 30 min). As anticipated, at the initial stage (0 min), the edges of all three electrodes displayed clarity and smoothness, indicating that Zn^2+^ plating had not yet commenced (refer to Figure 3d). However, with the progression of galvanizing time, clear and uneven zinc deposits emerged on the edges of the pristine Zn anode, even after 15 min of galvanizing. Furthermore, the uneven Zn deposition on the edges of the Zn anodes became more pronounced after 30 min of galvanizing. In contrast, this phenomenon was not noticed with the TiO_2_ and BTO‐coated anodes. The SEM images (Figure 3e) of the 30‐min Zn^2+^ plating clearly demonstrate an uneven surface on the pristine Zn anode (Figure 3e(i,ii)) due to dendrite growth, while no such dendrites were observed on the TiO_2_/Zn (Figure 3e(iii,iv)) and BTO/Zn (Figure 3e(v,vi)) anodes. This observation suggests that the coated samples effectively inhibited the formation of dendrites compared to the pristine anodes. Figure S4 (Supporting Information) shows the cross‐sectional SEM images of the anodes after 30 min of platting at 10 mA cm^−2^. Furthermore, for enhanced comprehension of the anodes' stability and endurance in the electrolytes, we immersed them in 3 m Zn(CF_3_SO_3_)_2_ electrolytes for six months. Following this period, the pristine Zn electrodes exhibited noticeable corrosion, whereas the treated electrodes showed minimal signs of corrosion, as illustrated in Figure S5 (Supporting Information). To gain further insights into the kinetics of Zn^2^⁺ plating/stripping and the behavior of coated anodes, we designed and tested special symmetric cells using a 1 × 1 cm^2^ cuvette design (Figure S6a,b, Supporting Information) over 100 h. Cross‐sectional SEM images, digital images, and EDS mappings were captured as shown in Figures S6 and S7. (Supporting Information) As expected, pristine Zn showed severe dendrite growth after 100 h of cycling, along with tip effects (Figure S6d, Supporting Information), and cross‐sectional SEM images clearly demonstrated uncontrolled dendrite growth on the pristine Zn surface. On the other hand, after cycling the TiO₂/Zn anode, an increase in coating thickness (Figure S6f,g, Supporting Information) and surface roughness was observed, likely due to dendrite growth between the TiO₂ and Zn interfaces, although much less pronounced than in the pristine Zn. Cross‐sectional images of BTO/Zn anodes before and after cycling (Figure S6h,i, Supporting Information) indicate a more uniform surface coating compared to Zn and TiO₂/Zn anodes, likely due to slower dendrite growth in BTO/Zn anodes. The inset digital image in Figure S6i (Supporting Information) shows that the white coating layer remains intact even after cycling, suggesting Zn^2^⁺ plating/stripping occurs at the BTO‐Zn interface. Furthermore, the EDS mappings of the anodes (Figure S7, Supporting Information) align with the findings in Figure S6 (Supporting Information), where significant surface roughness was observed along with the distribution of Zn elements in the cycled Zn anode. As expected, the EDS mappings of the BTO/Zn anode before and after cycling reveal a relatively uniform surface, indicating the dendrite‐suppressive behavior of the BTO‐coated Zn anode. Elemental mappings of Zn (Figure S7e, Supporting Information), Ba (Figure S7f, Supporting Information), Ti (Figures S7g–O and S7h, Supporting Information) after cycling further confirm that the signals from BTO are more prominent than those from Zn, suggesting that Zn^2^⁺ plating/stripping occurs at the BTO‐Zn interface.

This observation serves to reaffirm the enhanced stability of the coated Zn anodes compared to their pristine counterparts. Table S1 (Supporting Information) presents a comparison of the Zn plating/stripping performance in the current work with recently reported results for symmetric cells, which were fabricated using various surface coatings on Zn anodes.

**Figure 4:**
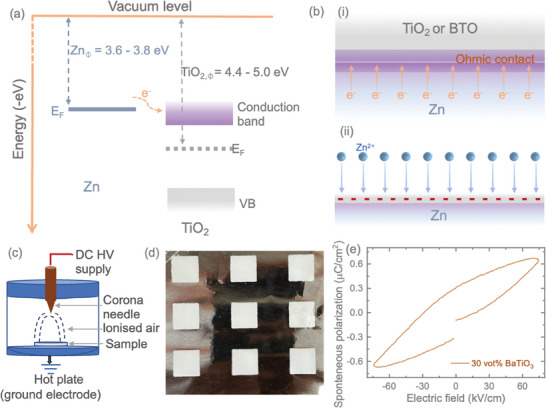
a) Illustration of electron flow from metallic Zn to TiO_2_, where $Zn_{\Phi }$ and *TiO*_2,Φ_ denote the work functions of Zn and TiO_2_, and *E_F_* represents the Fermi level. b) Depicts the formation of an ohmic contact interface between Zn and TiO_2_ (or BTO) and uniform electric flux distribution to promote uniform Zn^2+^ platting. c, d) Schematic representation of the corona poling technique and digital image of the printed 0–3 types BTO composite samples on an aluminum foil. e) Polarization‐electric field P–E) curve of the utilized BTO nanoparticles.

The effective suppression of dendrite formation and the achievement of uniform Zn^2+^ plating and stripping can be elucidated by examining the even distribution of electric fields on the surface of the reconstructed anodes. It is necessary to note that metallic Zn possesses a lower work function (3.6–3.8 eV) than TiO_2_ (4.4–5.0 eV).^[^ ^19^ ^]^ Generally, TiO_2_ is known to be an n‐type semiconductor. If the work function of an n‐type semiconductor is greater than that of a metal, ohmic contact formation occurs, facilitating the efficient flow of electrons without significant potential barriers. Thus, when Zn and TiO_2_ come into contact, electrons move from Zn to TiO_2_, establishing an ohmic contact interface (see **Figure** **4**a,b(i)). The negative charges accumulated at this interface attract Zn^2+^ ions, promoting improved Zn^2+^ diffusion kinetics and reducing the nucleation barrier for Zn, resulting in dendrite‐suppressive Zn deposition chemistry (see Figure 4b(ii)). On the other hand, BTO not only advances as an n‐type semiconductor forming an ohmic contact with Zn but also promotes the Maxwell‐Wagner effect and exhibits ferroelectric properties, i.e., the polarization of space charges under applied electric fields. Consequently, the strong electric flux generated on the surface of BTO nanoparticles provides more nucleation sites and enhances Zn^2+^ transport kinetics through “space charge polarization.” This further ensures uniform Zn^2+^ stripping/plating, reduces the overpotential, and improves long‐term cycling stability. To validate the ferroelectric properties of BTO nanoparticles, screen printing techniques were employed to create a thin layer, ≈20 microns thick, of 0–3 types BTO composite samples on an aluminum foil. The printed composite was then poled using a corona poling technique, as depicted in Figure 4c, at an elevated temperature of 120 °C and a voltage of 16 kV applied at the tip, ≈36.5 cm away from the sample (Figure 4d). Further details of this process can be found in the Experimental Section. The piezoelectric charge coefficient d_33_ was measured to be ≈10 pC N^−1^. Additionally, the Sawyer‐Tower technique was utilized to gather polarization versus electric field (PE) data, confirming the ferroelectric behavior. PE loops were recorded up to 75 kV cm^−1^ at room temperature, as shown in Figure 4e, with the hysteresis loop indicating remnant polarization and saturation polarization values of 0.31 and 0.66 µC cm^−^^2^, respectively. Based on these findings, it is hypothesized that BTO/Zn anodes exhibit a more robust and uniform field distribution, coupled with space charge accumulation, leading to a consistent flux distribution for Zn^2^⁺ deposition compared to TiO₂/Zn anodes. Consequently, this contributes to improved suppression of dendrite formation. Furthermore, the protective function of the BTO layer effectively mitigates corrosion and HER.

**Figure 5:**
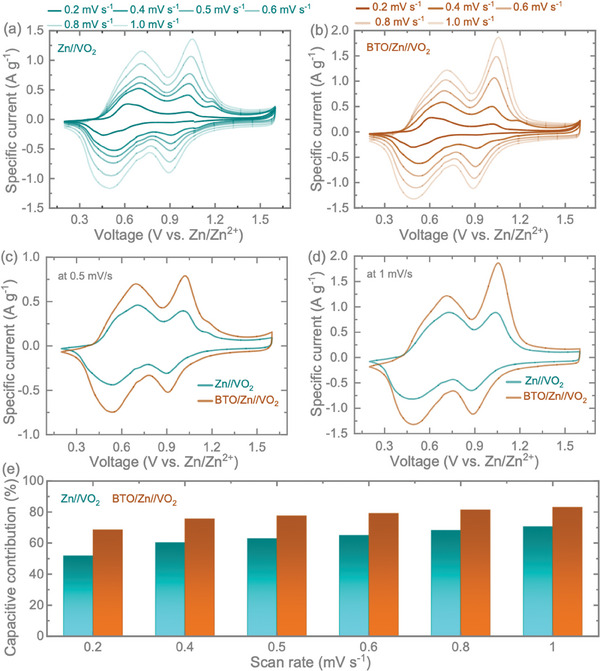
a,b) CVs of Zn//VO_2_ (B) and BTO/Zn//VO_2_ (B) at different scan rates. Comparative CV curves of Zn//VO_2_ and BTO/Zn//VO_2_ full cells tested at scan rates of c) 0.5 and d) 1 mV s^−1^. e) Capacitive contribution plot with respect to scan rate of the cells.

Subsequently, we conducted further investigations on BTO/Zn anodes for full‐cell tests paired with VO_2_ (B) cathodes to assess their charge storage performance and compared the results with pristine Zn anode counterparts. The synthesis of materials and respective characterization of VO_2_ (B) cathodes are detailed in the Supporting Information (refer to Figure S8, Supporting Information). The consistent cyclic voltammogram (CV) curves for both Zn//VO_2_ (**Figure** **5**a) and BTO/Zn//VO_2_ (Figure 5b) at different scan rates from 0.2 to 1.0 mV s^−1^ suggest stable and reversible charge storage performance of the cells. In Figure 5c,d, the comparative CV of Zn//VO_2_ and BTO/Zn//VO_2_ cells at scan rates of 0.5 and 1.0 mV s^−1^ within the voltage window of 0.2 to 1.6 V is presented. The CV curves revealed two pairs of redox peaks in both cells, corresponding to the two‐step intercalation of Zn^2+^ into VO_2_ (B) cathode. Specifically, the reduction/oxidation peak potentials for the Zn//VO_2_ full cell were 0.71/1.05 and 0.50/0.89 at 1.0 mV s^−1^. In the BTO/Zn//VO_2_ full cell, these peaks were observed at 0.71/1.06 and 0.49/0.89 at 1.0 mV s^−1^. Importantly, the redox reaction mechanism of VO_2_ (B) remained unchanged with the BTO coating on Zn but exhibited an increase in redox peak‐specific currents compared to the pristine Zn anode. This indicates that the artificial coating layer of BTO is effective in enhancing electrochemical reactivity and capacity, consequently improving peak current density.^[^ ^20^ ^]^ To further comprehend the improved charge storage performance after the BTO coating onto the Zn anode, we quantified the capacity contributions from capacitive‐controlled and diffusion‐controlled mechanisms. The process of charge storage can be analyzed in terms of capacitive‐controlled (*k*_1_ *v*) and diffusion‐controlled (*k*_2_ *v* ^0.5^) components, as indicated by the current at a fixed voltage relationship, *I* (*V*) =  *k*_1_ *v* + *k*_2_ *v* ^0.5^ or *I*(*V*)/ *v* ^0.5^ =  *k*_1_ *v* ^0.5^ + *k*_2_.^[^ ^21^ ^]^ Utilizing this equation, the overall capacitive contribution can be calculated to be ≈51.8% and 68.6% for Zn//VO_2_ and BTO/Zn//VO_2_ at a scan rate of 0.2 mV s^−1^. These values increase to 70.6% for Zn//VO_2_ and 83.0% for BTO/Zn//VO_2_ when the scan rate is raised to 1.0 mV s^−1^ (refer to Figure 5e). Consequently, with the incorporation of BTO on Zn, the overall charge storage in full cell configuration exhibits a synergistic improvement in capacitive contribution, resulting in enhanced rate capability for BTO/Zn//VO_2_ compared to Zn//VO_2_, see further.

**Figure 6:**
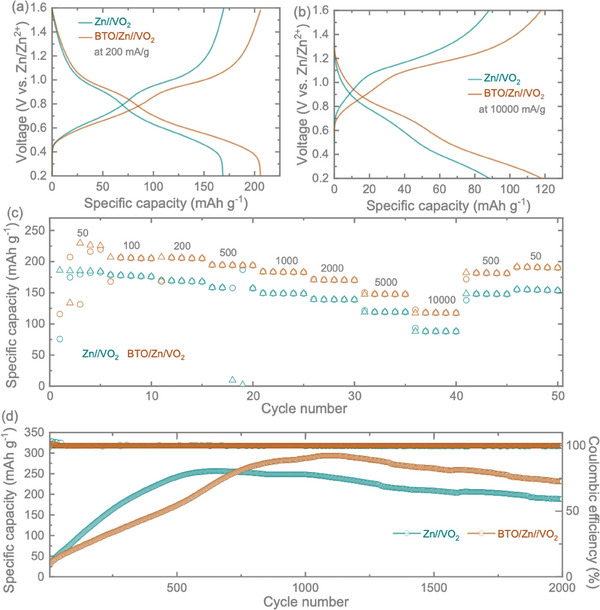
GDC tests for Zn//VO_2_ and BTO/Zn//VO_2_ full cell: a,b) GDC result at 200 and 10 000 mAh g^−1^, c) Rate tests performance of the cells, d) long‐time cycling result at1 A g^−1^.

Subsequently, we conducted Galvanostatic Discharge–Charge (GDC) performance assessments for Zn//VO_2_ and BTO/Zn//VO_2_ at various specific currents, ranging from 50 to 2000 mA g^−1^, within a voltage range of 0.2–1.6 V. Illustrated in **Figure** **6**a,b, the comparative GDCs at 200 and 10 000 mA g^−1^ reveal notable enhancements in charge storage performance for BTO‐coated anode‐based cells, spanning from low to high rates. For example, at a high specific current of 10 000 mA g^−1^ (Figure 6b), the measured specific capacities are 88 mAh g^−1^ for Zn//VO_2_ and 117 mAh g^−1^ for BTO/Zn//VO_2_. The substantial improvement, even at a high specific current, following the application of BTO onto the Zn anode may be attributed to enhanced Zn^2+^ platting/stripping kinetics contribution to charge storage, thereby enhancing rate capability. The rate test plot for Zn//VO_2_ and BTO/Zn//VO_2_ (Figure 6c) exhibits enhancements in specific capacities for BTO/Zn//VO_2_ compared to Zn//VO_2_ across various specific currents ranging from 50 to 2000 mA g^−1^. Additionally, Figure 6d presents long‐term cycling test results, revealing that the BTO/Zn//VO_2_ cells, formed by coating BTO onto the Zn anode, not only improve charge storage performance in terms of specific capacities but also enhance long‐term stability. After GDCs at 2000 mA g^−1^, the BTO/Zn//VO_2_ demonstrates a capacity of 231  mAh g^−1^ while Zn//VO_2_ exhibits a reduction to 186 mAh g^−1^. Consequently, our findings suggest that BTO coating on the Zn anode enhances rate capability and cycling stability by improving charge transport kinetics and inhibiting dendrite formation at the anode‐electrolyte interface. Moreover, the observed increase in the capacity of the materials during cycling is primarily due to the activation of active materials, a well‐documented phenomenon in aqueous Zn‐ion batteries. Table S2 (Supporting Information) shows a comparison between our performance results and those reported in other studies on ZIBs.

In this study, we developed a novel approach by creating an artificial SEI layer coated with high‐dielectric BTO on the zinc anode. This layer was applied using a simple spray‐coating method to reduce dendrite formation and prevent the HER. The high‐dielectric BTO layer forms both an ohmic contact interface and electric dipoles at the interface. The electric dipoles can be aligned by applying an external electric field, and during the charge–discharge process, the combined effect of the ohmic contact and electric dipoles helps to guide the migration of zinc ions. This alignment accelerates zinc‐ion kinetics and mitigates dendrite growth, as observed through *in situ* optical techniques. The BTO‐coated zinc anode demonstrates stable performance in terms of zinc stripping and plating, with a reduced voltage hysteresis of 91 mV for up to 750 h at a high current density of 5 mA cm^−2^ and a capacity of 5 mAh cm^−2^. Furthermore, the BTO/Zn electrode shows a lower activation energy of 22.6 kJ mol^−1^, higher resistance to corrosion, and enhanced oxygen reduction reaction (ORR) compared to the uncoated zinc anode. When used in full‐cell aqueous ZIBs, with a VO_2_ cathode, the BTO/Zn anode delivers excellent rate capability (117 mAh g^−1^ at 10 A g^−1^) and extended cycle life, retaining 231 mAh g^−1^ after 2000 cycles. This work contributes to the development of a stable zinc metal anode for high‐performance aqueous zinc‐ion batteries.

## Conflict of Interest

The authors declare no conflict of interest.

## Supporting information



Supporting Information

## Data Availability

The data that support the findings of this study are available from the corresponding author upon reasonable request.
