# Supplementary material for: Ferroelectric Interfaces for Dendrite Prevention in Zinc‐Ion Batteries
Source: Small. 2024 Sep 15;20(49):2403555. doi: 10.1002/smll.202403555 (PMC11618717; doi:10.1002/smll.202403555)
Supplement: Supplementary file 1 — Supporting Information [file SMLL-20-2403555-s001.pdf]

NANO · MICRO
small

Supporting Information

for *Small*, DOI 10.1002/smll.202403555

Ferroelectric Interfaces for Dendrite Prevention in Zinc-Ion Batteries

*Xueqing Hu, Bastola Narayan, Nibagani Naresh, Iman Pinnock, Yijia Zhu, Xiaopeng Liu, Tianlei Wang, Bing Li, Ivan P. Parkin and Buddha Deka Boruah**

# Supporting Information
# for
# Ferroelectric Interfaces for Dendrite Prevention in Zinc-ion Batteries

Xueqing Hu,[1] Bastola Narayan,[2] Nibagani Naresh,[1] Iman Pinnock,[1] Yijia Zhu,[1] Xiaopeng Liu,[1] Tianlei Wang,[3] Bing Li,[1] Ivan P. Parkin,[3] Buddha Deka Boruah[1,*]

[1]Institute for Materials Discovery (IMD), University College London (UCL), London WC1E 7JE, UK

[2]Department of Mechanical Engineering, University of Bath, Bath, BA2 7AY, UK

[3]Department of Chemistry, University College London (UCL), London WC1H 0AJ, UK

Corresponding author: Dr. Buddha Deka Boruah: b.boruah@ucl.ac.uk

**Experimental Section**

*Chemicals and materials:* Ammonium metavanadate ($NH_4VO_3$, Fisher Scientific), formic acid ($CH_2O_2$, VWR Life Science) and Ethanol absolute (Fisher Scientific) were used to prepare of $VO_2$(B) powder. 1-Methyl-2-pyrrolidinone (NMP, Sigma Aldrich, 99.5%) was used as solvent which mixed with BTO and $TiO_2$ powder for spray coating. Poly(vinylidene fluoride) Powder (Alfa Aesar) was used as a binder to increase viscosity of cathode mixture. Barium titanium oxide, 99% (Thermo scientific), Titanium(IV) oxide rutile (Sigma Aldrich) were mixed with NMP in ratio 9 : 1 for spray coating.

*Characterization:* X-ray diffraction (XRD) measurements were carried out using an Aeris Research Benchtop X-ray diffractometer with Cu Kα radiation (λ = 0.15418 nm). Morphological analyses were performed using a Carl Zeiss SIGMA HD VP field emission scanning electron microscope in combination with an Oxford Aztec ED X-ray analysis system. Optical images were taken with a Zeiss AXIO Scope.A1 Trinocular Pathology microscope using a 2.5x objective lens. X-ray photoelectron spectroscopy (XPS) data were collected using a Thermo Fisher K Alpha instrument. The thickness of electrode was detected using Bruker Dektak XT stylus profilometer.

*Polarization-electric field measurement:* A custom-made corona poling set put is used to poled screen printed BTO based composite at 110 °C at 15 kv for 30 minutes. Sample was place under corona needle tip which is 45 mm far from the sample place. A Berlincourt piezometer (Piezotest, PM300, Singapore) at 97 Hz was used to measure longitudinal coefficient ($d_{33}$). Polarization-electric field (P-E) loops were measured with a Precision Premier II Loop Tracer (Radiant Technologies) at the room temperature.

*Anode preparation:* Initially, 0.18mg of BTO and $TiO_2$ were mixed with 0.02mg Poly(vinylidene fluoride) Powder (PVDF) separately. After that, 0.2mg mixture were added into 20ml 1-Methyl-2-pyrrolidinone (NMP) separately. Then, both of 20ml mixture were spray coated on top of hot plate at 140 °C. The thickness of electrodes were detected using Bruker Dektak XT stylus profilometer (10μm).

*Synthesis of $VO_2$ nanowires:* Initially, in 60ml of deionized water, 0.7g of Ammonium metavanadate ($NH_4VO_3$) was dispersed via magnetic stirring. Once the $NH_4VO_3$ powder was fully dispersed, 4.8 mL of formic acid ($CH_2O_2$) was added into the solution, which was magnetically stirred for 1 hour. After mixing, the solution was transferred to Teflon-liners

which held 30 mL, and placed in the microwave reactor for 8 hours and 40 mins (520 mins) at 200°C. The reactor used was time limited to 3 hours and 15 minutes, so the machine was ran 3 times to ensure the full 520 mins was achieved. Once finished, the product was washed with ethanol and deionized water, and dried at 70°C overnight.

*In-situ optical imaging:* In-situ optical images were recorded using a ZEISS AXIO Scope.A1. Acrylic molds were employed to streamline the in-situ optical imaging procedure. These molds are composed of a one-centimeter thick acrylic sheet engraved with an H-shaped pattern, featuring a width and depth of 0.5 cm. The electrode sheets are placed within the groove on the longer side of the mold, while the two sides of the mold are interconnected using an electrolyte.

*Symmetric cell test:* The components for the symmetrical cell (CR2032 coin cell) were obtained from Cambridge Energy Solutions. The separator employed was a Waterman Grade GF/B Glass Microfiber Filter Binder Free 55mm. Additionally, a 3M electrolyte was prepared using zinc trifluoromethanesulfonate ($Zn(CF_3SO_3)_2$). The symmetrical cells were assembled with 150 µl of the electrolyte. Voltage profiles were acquired using the CT-4000 Battery Testing System at various different rates, including 0.1, 0.2, 0.5, 1, and 5 mA $cm^{-2}$. Linear-sweep-voltammetry (LSV) tests were conducted at a rate of 5 mV $s^{-1}$ during the second cycle, and Tafel plots were generated by measuring symmetric cells at a scan rate of 1 mV $s^{-1}$. Additionally, Electrochemical Impedance Spectroscopy (EIS) was carried out on the symmetric cells at various temperatures, including 25 °C, 30 °C, 40 °C, 50 °C, 60 °C, and 65 °C, all within the frequency range of 10 mHz to 100 KHz.

*Full cell test:* Both full and half cells were constructed following an identical procedure, utilizing the same electrolyte and components. Carbon paper served as the current collector for the cathode, while $VO_2$ nanofibers were employed as the active material. The cathode material comprised a blend of $VO_2$ nanofibers, super P, and PVDF in a ratio of 70:20:10. To ensure a uniform distribution on the carbon paper's surface, a mixture of 0.1g of powder and 12 ml of NMP was applied using a Doctor Blade. Galvanostatic discharge/charge (GDC) cycling tests and cyclic voltammetry (CV) profiles were performed on the MPG-200 Biologic battery test system. Electrochemical impedance spectroscopy (EIS) measurements were recorded in the frequency range of 10 mHz to 100 KHz using an Autolab (Metrohm) instrument.

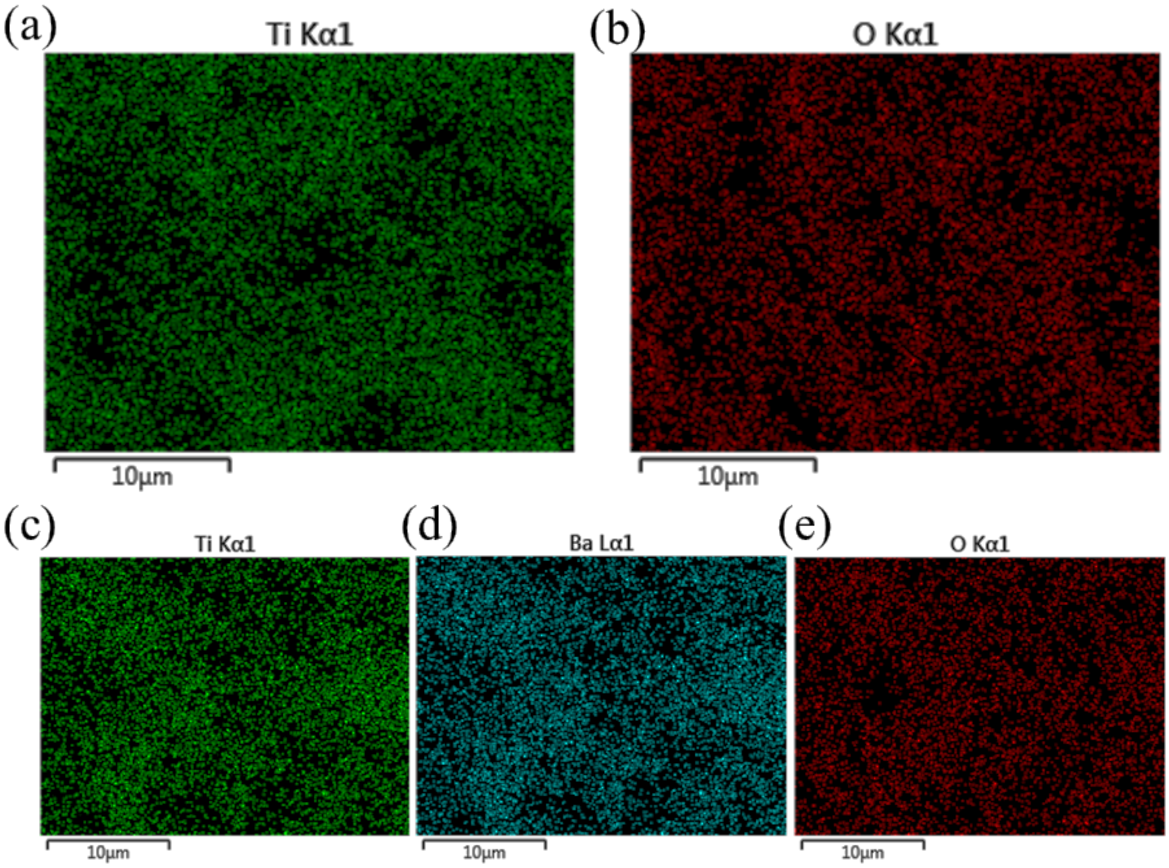


***Figure S1.*** *EDS mapping of the (a) Ti and (b) O elements of $TiO_2$ nanoparticles. (c) Ti, (d) Ba and (e) O elemental mapping of BTO particles.*

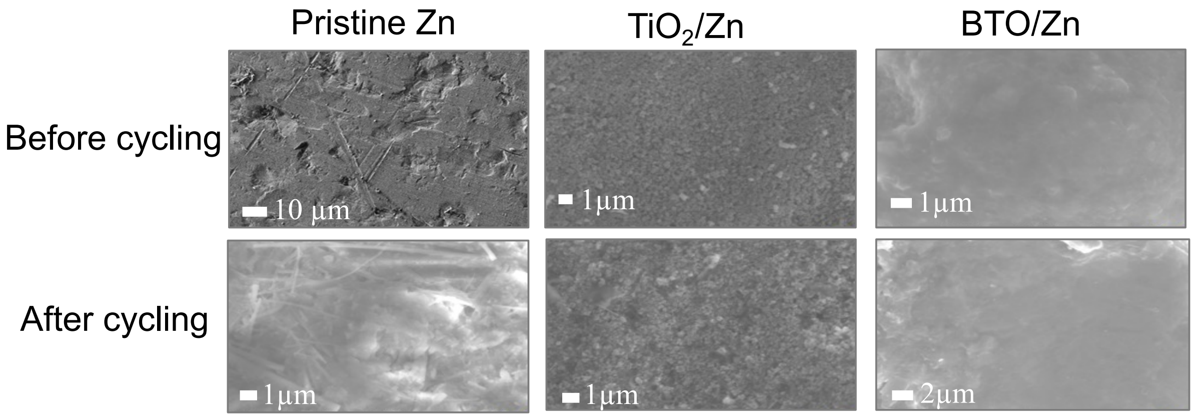


***Figure S2.*** *Post-mortem SEM images of the cycled electrodes at 1 mA cm$^{-2}$: Zn, $TiO_2$/Zn, and BTO/Zn.*

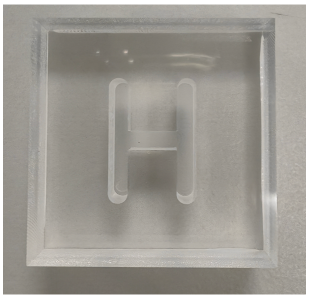

***Figure S3.*** *Digital image of the optical cell for in-situ optical imaging of zinc dendrite growth.*

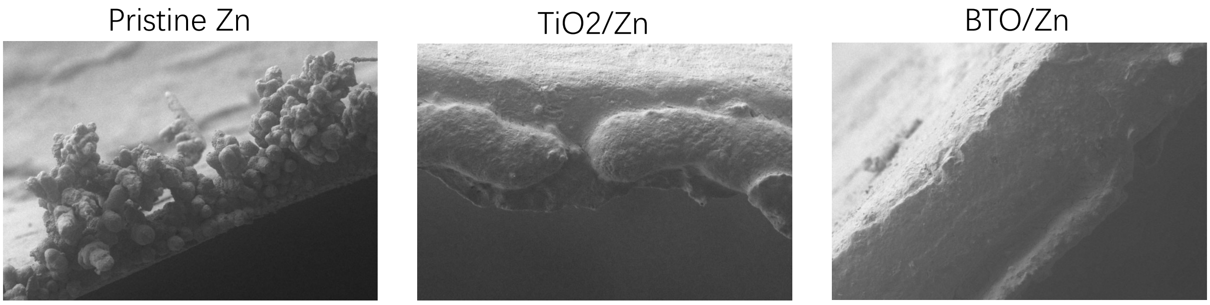


***Figure S4.*** *Cross-sectional SEM images of the anodes after 30 minutes of platting at 10 mA cm*$^{-2}$.

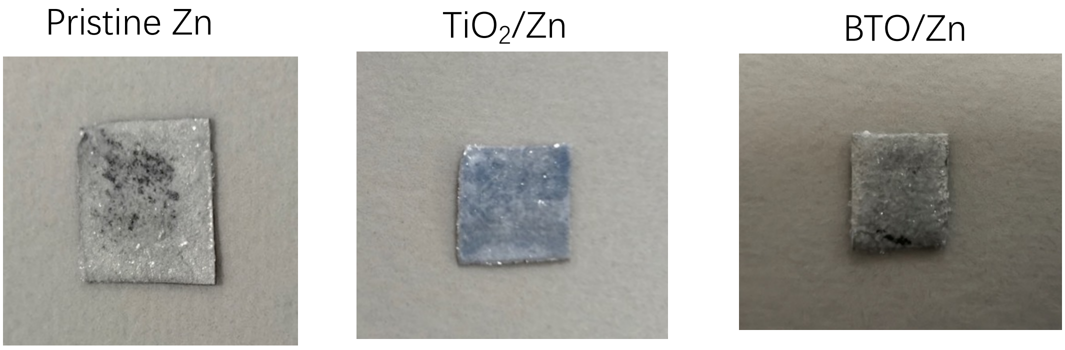


***Figure S5.*** *Pictures of the anodes after immersion into the 3M* $Zn(CF_3SO_3)_2$ *electrolytes for six months.*

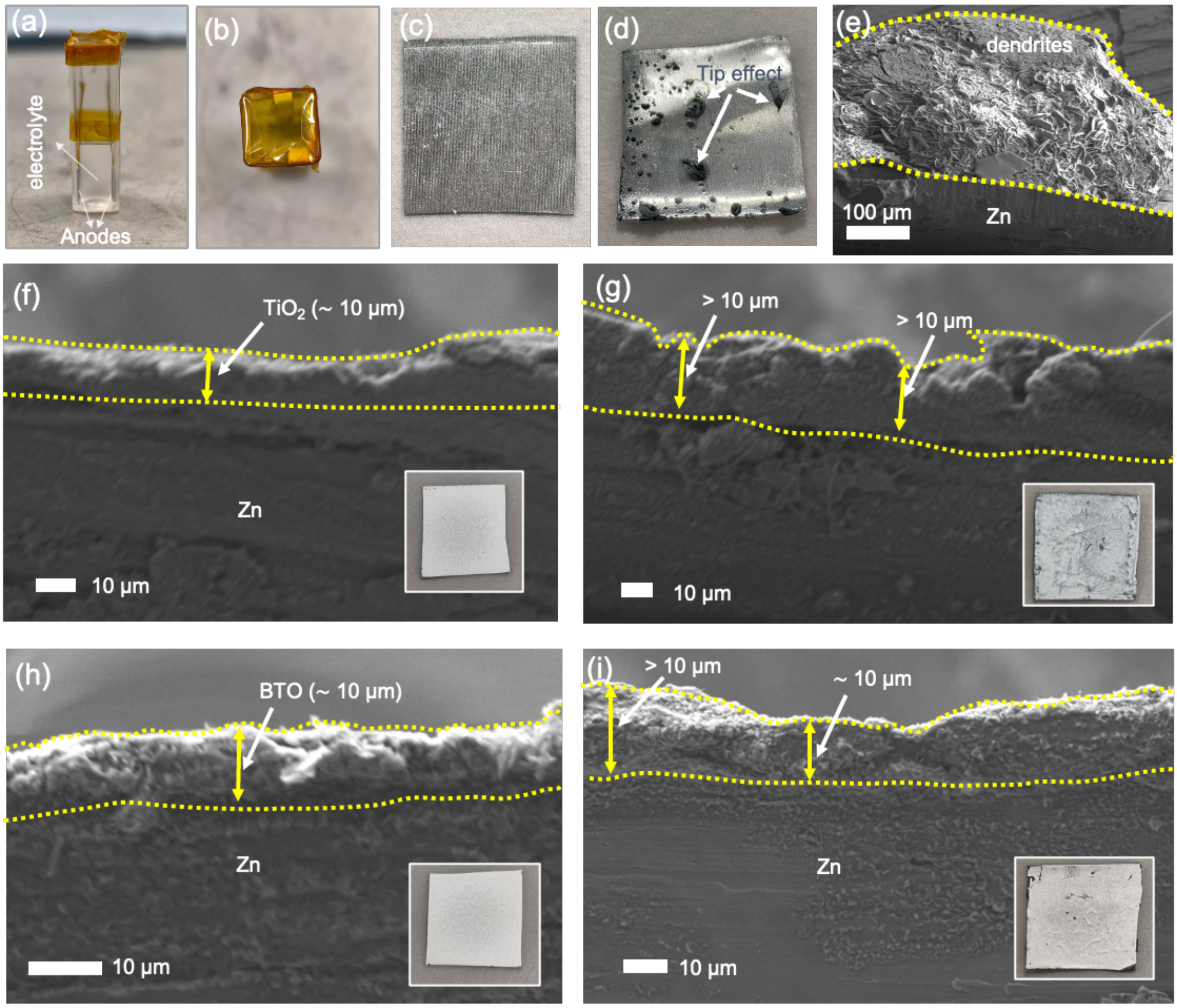


***Figure S6.*** *(a, b) Digital images of a symmetric cells tested in 1 × 1 cm² cuvette cell. These cells were tested for 100 hours to analyze changes in anodes morphologies and $Zn^{2+}$ stripping/plating kinetics. (c, d) Digital images of the pristine Zn anode before and after cycling, showing significant dendrite growth and large black spots due to the tip effect. (e) Cross-sectional SEM image confirming severe dendrite growth on the Zn anode after 100 hours of cycling. (f, g) Cross-sectional SEM image of a $TiO_2$ (~10 μm)/Zn anode, with an inset digital image of the $TiO_2$/Zn anode. After cycling, an increase in the coating thickness and surface roughness is observed, likely due to dendrite growth between the $TiO_2$ and Zn interfaces. The inset shows the cycled $TiO_2$/Zn anode. (h, i) Cross-sectional images of BTO/Zn anodes before and after cycling, indicating a more uniform surface coating compared to Zn and $TiO_2$/Zn anodes, likely due to slower dendrite growth in BTO/Zn anodes. The inset digital image shows that the white coating remains even after cycling, suggesting $Zn^{2+}$ plating/stripping occurs between the BTO and Zn interfaces.*

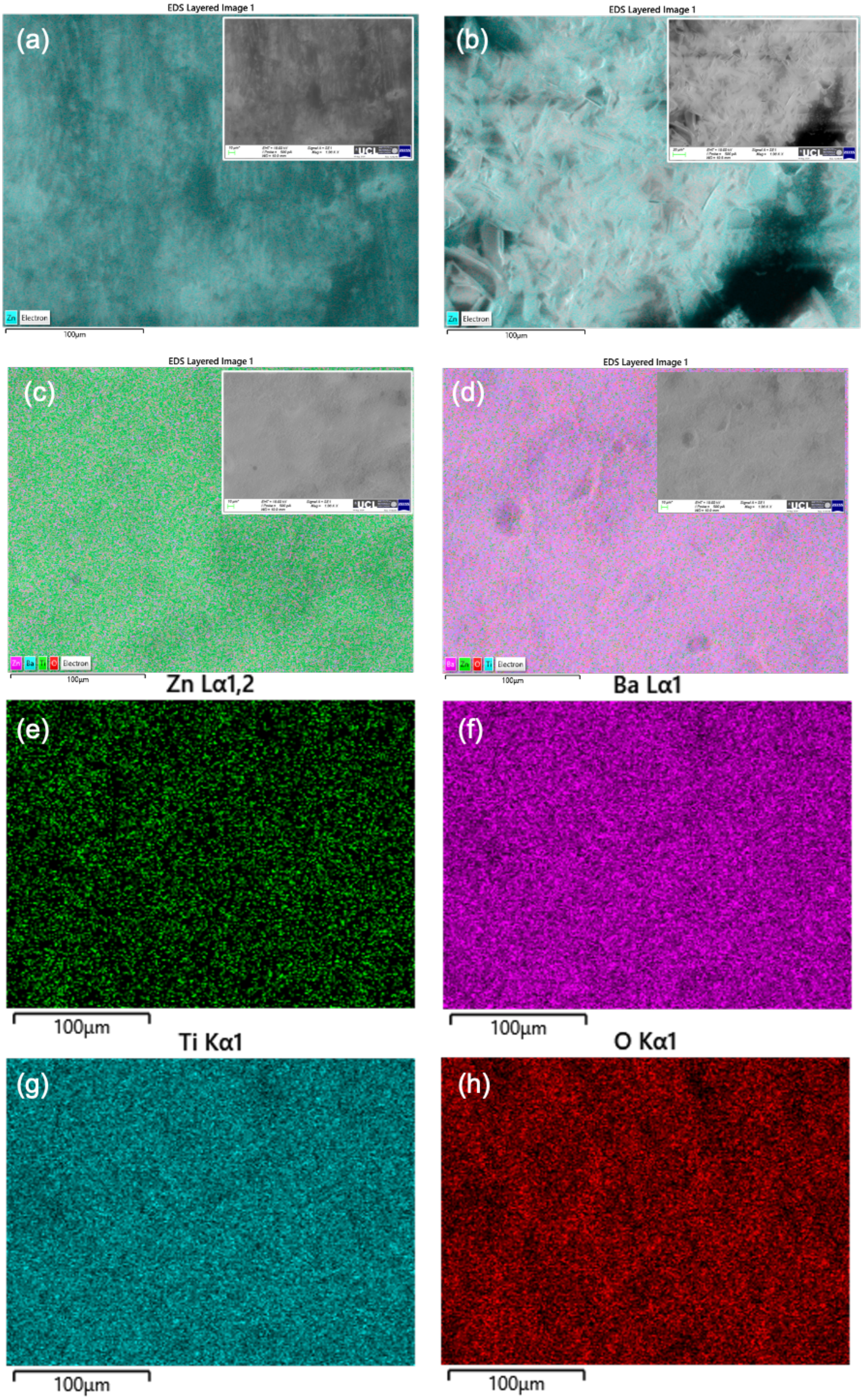


***Figure S7.*** *EDS mappings of the Zn anode (a) before and (b) after cycling, as shown in* ***Figure S6****. The insets display corresponding SEM images that further confirm dendrite growth and the*

*distribution of Zn elements across the samples. (c, d) EDS mappings of the BTO/Ti anode before and after cycling reveal a relatively uniform surface, indicating the dendrite-suppressive behavior of the BTO-coated Zn anode. Elemental mappings of (e) Zn, (f) Ba, (g) Ti, and (h) O after cycling further confirm that the signals from BTO are more prominent than those from Zn, suggesting that $Zn^{2+}$ plating/stripping occurs at the BTO-Zn interface.*

***Table S1.*** *Galvanostatic Zn plating/stripping performance achieved in current work compared with recently reported Zn//Zn symmetric cells fabricated suing different surface coating.*

| Electrode | Electrolyte | Current density [mA cm$^{-2}$] | Areal capacity [mAh cm$^{-2}$] | Voltage hysteresis [mV] | Ref |
|---|---|---|---|---|---|
| **BTO/Zn//$VO_2$** | 3M $Zn(CF_3SO_3)_2$ | 1 | 1 | 47 | |
| **$ZrO_2$/Zn//Zn** | 2M $ZnSO_4$ | 5 | 1 | 110 | [1] |
| **Gel-Ma/Zn//Zn** | 1M $(CF_3SO_3)_2Zn$ | 1 | 1 | 260 | [2] |
| **$Nb_2O_5$/Zn//Zn** | 2M ZnSO4 | 1 | 0.5 | 97 | [3] |
| **CB-NFC/Zn//Zn** | 2M $ZnSO_4$+0.2M $MnSO_4$ | 5 | 1.25 | 160 | [4] |
| **NiCo-LDH/Zn//Zn** | 2M $ZnSO_4$+0.2M $MnSO_4$ | 0.5 | 0.5 | 120 | [5] |

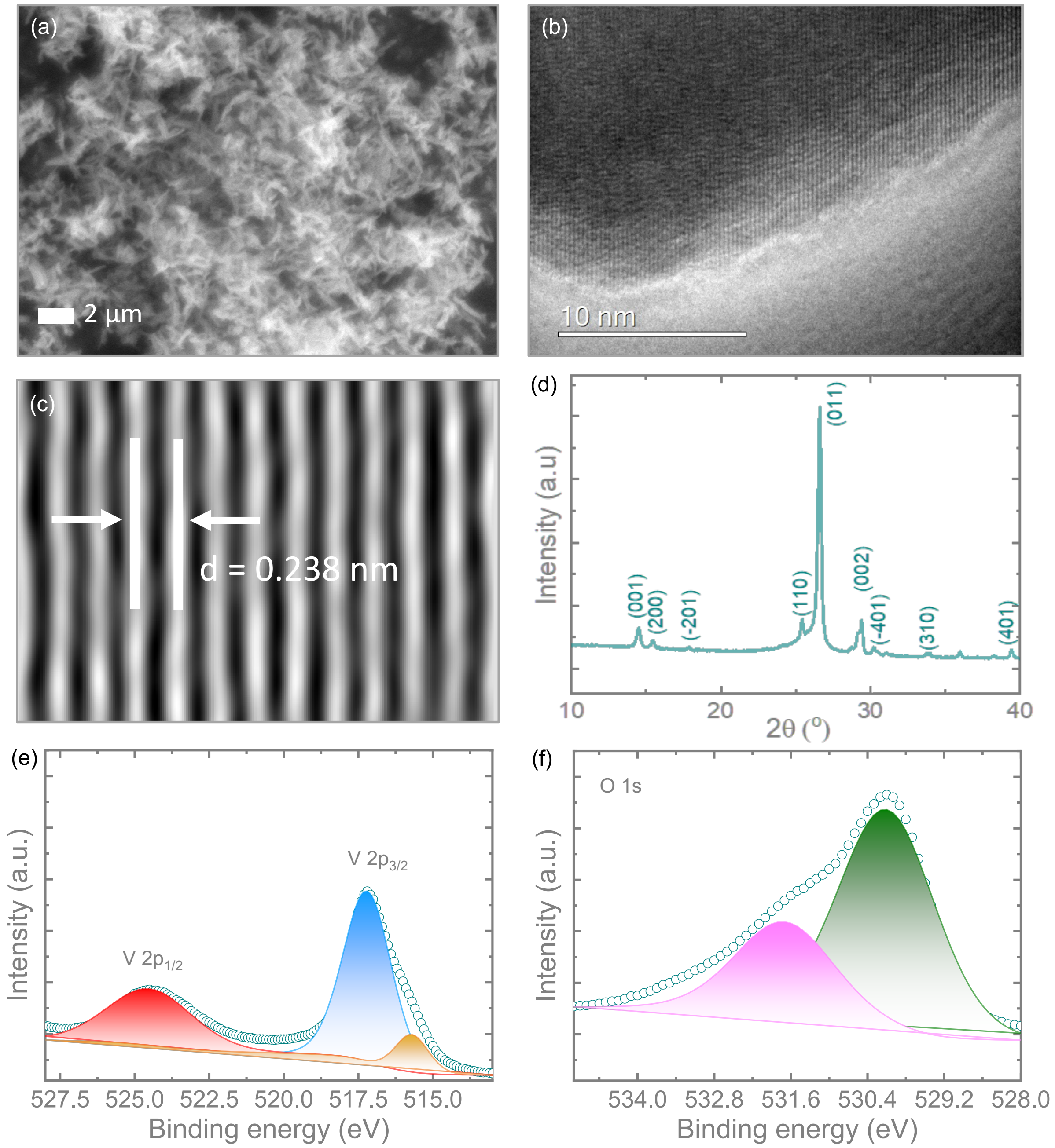


***Figure S8.*** *(a, b) SEM and HRTEM images of used $VO_2$(B). (c) Calculation of interplanar spacing of 0.238 nm that corresponds to (401) planes of $VO_2$(B). (d) XRD pattern of $VO_2$(B) can be indexed to the monoclinic $VO_2$ (B) phase with space group of C2/m. XPS of $VO_2$(B): (e) V2p and (f) O1s.*

**Figure S8a** presents the SEM image of a used $VO_2$(B) cathode, with the corresponding HRTEM image displayed in **Figure S8b**. The calculated interplanar spacing of 0.238 nm (**Figure S8c**), consistent with the (401) planes of the monoclinic $VO_2$(B) structure, is further validated by the XRD pattern shown in **Figure S8d**, indicating that this phase belongs to the

C2/m space group of monoclinic $VO_2$(B).The X-ray photoelectron spectrum (XPS) was utilized to examine the valence states of V and O in $VO_2$, as depicted in Figure. The V 2P spectrum exhibits two prominent peaks, namely V $2P_{1/2}$ and V $2P_{3/2}$, positioned at 524.44 eV and 517.21 eV, respectively. The V $2P_{3/2}$ spectrum was resolved into $V^{4+}$ (515.75 eV) and $V^{5+}$ (517.23 eV) states using Lorentzian-Gaussian fitting. The occurrence of $V^{4+}$ in $VO_2$ could be attributed to V(IV) doping, which leads to an increased lattice constant and a reduction in diffusion resistance, consequently enhancing electronic conductivity. The O 1S spectrum was resolved into $V^{4+-}$O (531.72 eV) and $V^{5+}$-O (530.14 eV) components, respectively.

***Table S2.*** *Contrasting the retained specific capacity following cycling in the present study with findings from prior research.*

| Electrode | Electrolyte | Current density [A $g^{-1}$] | Specific Capacity [mAh $g^{-1}$] | Columbic Efficiency [%] | Ref |
|---|---|---|---|---|---|
| **BTO/Zn//$VO_2$** | 3M $Zn(CF_3SO_3)_2$ | 1 | 231 (at 2000 cycle) | 99.9 | This work |
| **P-Zn//$VO_2$** | 2 M $ZnSO_4$ + 0.1 M $MnSO_4$ | 1 | 236.1 | 99.9 | [6] |
| **ZnO/Zn//$VO_2$** | 3 M $Zn(CF_3SO_3)_2$ | 0.2 | 121 | 99.9 | [7] |
| **$TiSe_2$/Zn//$VO_2$** | 2M $ZnSO_4$ | 0.2 | 128 | 99.9 | [8] |
| **W-$VO_2$/ Zn//$VO_2$** | 3 M $Zn(CF_3SO_3)_2$ | 0.1 | 281.3 (after 100 cycles) | 99.9 | [9] |
| **ZS-CZ-0.05** | 2M $ZnSO_4$ | 1 | 221 | 99.8 | [10] |

*References*

[1] P. Liang, et al. Adv. Funct. Mater. 2020, 30, 1908528.
[2] J. Shin, et al. Adv. Energy Mater. 2021, 11, 2100676.
[3] H. Liu, et al. Adv. Sci. 2021, 8, 2102612.
[4] A. Wang, et al. J. Colloid and Interface Science. 2020, 577, 256-264.
[5] C. Ma, et al. Chem. Eng. J. 2022, 429, 132576.
[6] Zhang, Q,et al. Chem. Eng. J. 2023, 474, 145981.
[7] Yujun Zhou, et al. Colloid and Interface Science. 2023, 630, 676-684.
[8] Wen Li, et al. Nano Energy. 2022, 93, 106896.
[9] Mei Li, et al. ACS Sustainable Chemistry & Engineering. 2021, 9, 42, 14913-14201.
[10] Yang Gao, et al. Journal of Energy Chemistry. 2023, 84, 62-72.
